# Identification of common coexpression modules based on quantitative network comparison

**DOI:** 10.1186/s12859-018-2193-3

**Published:** 2018-06-13

**Authors:** Yousang Jo, Sanghyeon Kim, Doheon Lee

**Affiliations:** 1 Bio-Synergy Research Center, Daejeon, 34141 South Korea; 2 Department of Bio and Brain Engineering, Korea Advanced Institute of Science and Technology (KAIST), Daejeon, 34141 South Korea; 3 Brain Research Laboratory, Stanley Medical Research Institute, Rockville, MD 20850 USA

**Keywords:** Network comparison, Network similarity, Coexpression network, Aging, Huntington’s disease

## Abstract

**Background:**

Finding common molecular interactions from different samples is essential work to understanding diseases and other biological processes. Coexpression networks and their modules directly reflect sample-specific interactions among genes. Therefore, identification of common coexpression network or modules may reveal the molecular mechanism of complex disease or the relationship between biological processes. However, there has been no quantitative network comparison method for coexpression networks and we examined previous methods for other networks that cannot be applied to coexpression network. Therefore, we aimed to propose quantitative comparison methods for coexpression networks and to find common biological mechanisms between Huntington’s disease and brain aging by the new method.

**Results:**

We proposed two similarity measures for quantitative comparison of coexpression networks. Then, we performed experiments using known coexpression networks. We showed the validity of two measures and evaluated threshold values for similar coexpression network pairs from experiments. Using these similarity measures and thresholds, we quantitatively measured the similarity between disease-specific and aging-related coexpression modules and found similar Huntington’s disease-aging coexpression module pairs.

**Conclusions:**

We identified similar Huntington’s disease-aging coexpression module pairs and found that these modules are related to brain development, cell death, and immune response. It suggests that up-regulated cell signalling related cell death and immune/ inflammation response may be the common molecular mechanisms in the pathophysiology of HD and normal brain aging in the frontal cortex.

**Electronic supplementary material:**

The online version of this article (10.1186/s12859-018-2193-3) contains supplementary material, which is available to authorized users.

## Background

### Coexpression analysis and biological network comparisons

Gene expression profiling is one of the best windows that shows a snapshot of cellular activity. It shows what activity is promoted and what activity is inhibited in the certain condition [1]. Therefore, there have been numerous approaches to understand gene expression data properly and they have used various traits of gene expression data [2]. For instance, statistical significance and fold-change of each gene have been widely used to find the difference between cohorts [3]. However, these traits only focused on single gene so they were sensitive to noise [4]. As a consequence, coexpression analysis that provides more robust modular marker has risen [5].

Briefly, coexpression analysis is the method to extract gene pairs that have positively or negatively coexpressed [6]. And ‘coexpressed genes’ are mathematically defined as gene pairs which have a correlation above the certain threshold and they are known as genes which related to similar biological functions [6]. Also, coexpressed genes in certain condition are not separated but closely interact with each other and are called ‘coexpression module’. Coexpression module is considered as a robust modular molecular marker. Therefore, coexpression profile of gene expression data can be represented as network form consists of genes as nodes and coexpression as edges and this network refers to ‘coexpression network’ [7]. Therefore, there have been coexpression studies which compare coexpression networks in different conditions such as species, [8] tissue, [9] and disease states [10].

Among coexpression analysis, finding common coexpression profiles between different samples can be an effective way to understand diseases or biological processes. For example, we can infer molecular mechanism of complex disease using common coexpression networks from well-known other diseases. Many previous studies simply extract overlapping nodes and edges as common coexpression networks because they dealt with two or fewer networks. However, if there are multiple sample groups or we perform modular analysis, we should deal with several coexpression networks. Quantitative network comparison can clearly provide similar network pairs among multiple coexpression networks and it leads to finding common coexpression profiles among sample groups or modules.

For other biological networks, there are various network comparison methods. Network comparison methods for other networks can be divided into two categories: alignment-based methods and alignment-free methods [11]. Alignment-based methods were developed to align two or more homologous networks such as protein-protein interaction networks. They assumed networks in the query that networks diverged from the same network and they have homologous regions [12]. Due to this assumption, network alignment-based methods align genes in a similar network topology. However, coexpression analysis deals with genes from same species so exact matching of networks (finding same subnetwork) is more suitable than network alignment (finding similar subnetwork). The othercategory of methods is the alignment-free method and it is divided into graphlet-based methods and functionality-based methods. Graphlet-based methods count small subgraphs called ‘graphlet’ and measures network similarity based on graphlet frequency. However, these methods only consider topological information of graphlets and blind information of each genes. It leads inappropriate comparison for coexpression network. Functionality-based methods utilize functional enrichment information of networks. So they can be used for any networks consists of genes but it provides only indirect comparison. Therefore, we concluded that there is no proper network comparison for coexpression network.

### Huntington ‘s disease and brain aging

Huntington’s disease (HD), also known as Huntington’s chorea is neurological disorder famous for its autosomal dominant inheritance. Previous findings suggest that HD allele in chromosome four may cause the toxic gain of function for HD-related genes such as Huntingtin (HTT) and it leads to massive neuronal cell death [13]. Consequently, HD patients suffer from uncontrolled movements, abnormal body postures, and changes in behavior, emotion, judgment, and cognition. However, the molecular mechanism of HD is poorly understood so there is no cure to slow, stop, or reverse HD yet [14].

Unlike many neurological diseases, HD is an inherited disease. People who have the HD allele can have disease onset anytime in their life (especially at age 30–50) and usually die within 15–20 years [15]. In other words, patients can be suffer from HD regardless of their age.

Interestingly, many brain imaging studies suggested that functional deficits in HD patients are strongly correlated with aging-related functional deficits such as dopamine receptors [16]. Since HD can arise in any age, these HD-functional deficits are not a consequence of aging. Therefore, we can infer that there may be common or similar mechanism between HD and brain aging. We focus on the similarity in molecular mechanism between HD and brain aging and we tried to find similar molecular modules between HD and brain aging based on quantitative coexpression analysis.

In this study, we applied quantitative coexpression analysis to find common molecular features between HD and brain aging. We proposed two similarity measures for quantitative comparison of coexpression modules. We then showed the validity of these measures and determined the threshold similarity of similar coexpression module pairs using known coexpression networks. Using these similarity measures and thresholds, we quantitatively compared HD-specific and aging-related coexpression modules and found similar HD-aging coexpression module pairs. We inferred possible common molecular mechanisms from similar HD-aging coexpression module pairs.

## Methods

This study is divided into two parts. In the first part, we proposed quantitative similarity measures for coexpression networks and performed validation of these measures. We also evaluated the threshold value of similar modules. In the second part, we extracted HD-related coexpression modules and aging-related coexpression modules and compared these modules quantitatively based on coexpression network similarities. Then we found similar HD – aging module pairs and interpreted their biological significance.

### Coexpression network similarity measures

Coexpression networks can be interpreted as weighted networks consisting of nodes (genes) and edges (degree of coexpression between two genes). Therefore, we can define similarity between two coexpression networks based on node consistency (‘how many common genes they have’) and edge consistency (‘how many coexpressions they share’). To utilize both sets of information, we developed node-based similarity adjusted by edge information, COEXsim and employed fuzzy set similarity as edge-based similarity.

#### Node-based similarity: COEXpression-based similarity (COEXsim)

**Fig. 1:**
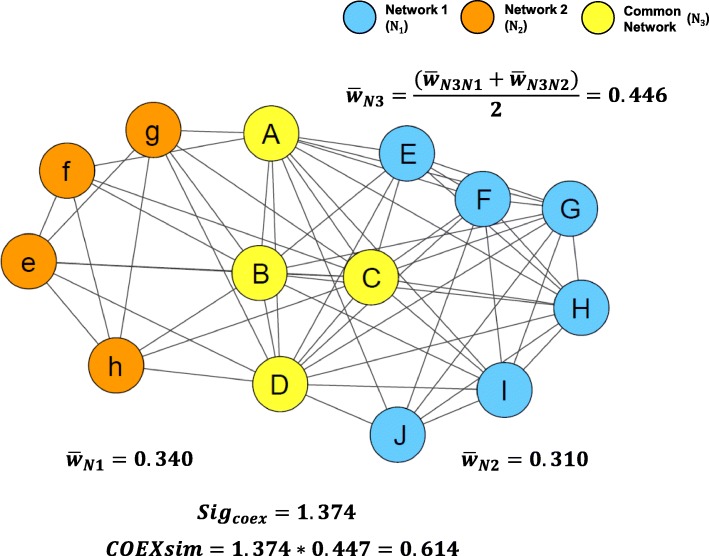
The Concept of COEXsim. COEXsim is determined by relative size and relative degree of coexpression of common subnetwork (N_3_) of two networks

We developed Coexpression-based network similarity (COEXsim) to quantify the similarity between two coexpression networks based on their node consistency. As a method to quantify network similarity, COEXsim has the following two features: (1) It extracts common subnetwork from two networks to measure consistency between two networks, (2) It shows coexpression significance of common subnetwork relative to two networks to reflect the nature of coexpression network. Therefore, we defined COEXsim of two networks

1 $$ COEXsim= Siz{e}_{rel}\ast Si{g}_{coex} $$

as relative size of common subnetwork (*Size*_*rel*_) adjusted by coexpression significance (*Sig*_*coex*_) of two networks (Fig. 1). ‘Common subnetwork’ refers to the subnetwork consists of genes and edges that are present in both networks and we extracted common subnetwork by exact matching of nodes and edges.

We defined relative size of common network as node size of common network normalized by node sizes of two networks,

2 $$ {Size}_{Rel}=\frac{N{S}_{common}}{\sqrt{NS_{N1}}\sqrt{NS_{N2}}} $$

where *NS*_*N*1_, *NS*_*N*2_, *NS*_*common*_ are node sizes of network1, network2, and common network respectively.

Also, we defined coexpression significance as relative coexpression power of common subnetwork relative to that of two networks. In coexpression network, coexpression power of the network is represented by weights of edges. Hence, we defined coexpression significance between two networks as relative value of mean weight of common subnetwork to that of two networks

3 $$ {Sig}_{coex}=\frac{{\overline{w}}_{N3}}{\sqrt{{\overline{w}}_{N1}}\sqrt{{\overline{w}}_{N2}}}=\frac{\left({\overline{w}}_{N3N1}+{\overline{w}}_{N3N2}\right)}{2\sqrt{{\overline{w}}_{N1}}\sqrt{{\overline{w}}_{N2}}},\kern0.75em where\ {\overline{w}}_{N3}=\frac{\left({\overline{w}}_{N3N1}+{\overline{w}}_{N3N2}\right)}{2} $$

where ${\overline{w}}_{N1},{\overline{w}}_{N2},{\overline{w}}_{N3N1},{\overline{w}}_{N3N2}$ are mean weights of network1, network2, common subnetwork from network1 and network2 weight values, respectively.

From formula (1), (2), (3), COEXsim is mathematically represented as

4 $$ COEXsim=\frac{N{S}_{common}}{\sqrt{NS_{N1}}\sqrt{NS_{N2}}}\times \frac{\left({\overline{w}}_{N3N1}+{\overline{w}}_{N3N2}\right)}{2\sqrt{{\overline{w}}_{N1}}\sqrt{{\overline{w}}_{N2}}} $$

Therefore, we can understand COEXsim as a node-based similarity of two coexpression networks adjusted by edge consistency. COEXsim is increased when two networks shares more nodes or common subnetwork has more powerful coexpression (weight) than other parts of networks.

#### Edge-based similarity: Fuzzy set-based similarity

**Fig. 2:**
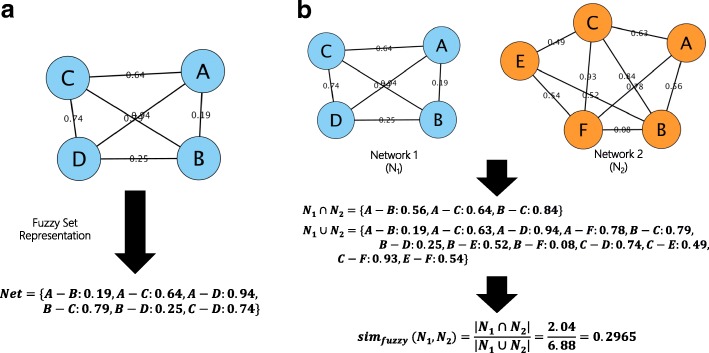
The Concept of Fuzzy Set Similarity. **b** Coexpression network can be interpreted as fuzzy set. **b** The similarity between two coexpression networks can be obtained by measuring fuzzy set similarity between two fuzzy sets

In COEXsim, we focused on the number of overlapped genes of two coexpression networks. However, the consistency in gene expression profile is also important information and weighted edges in coexpression network reflect coexpression between genes so we defined edge-based similarity. Weighted networks can be represented as fuzzy sets that edges are elements and weights are corresponding degrees of membership (Fig. 2a). Therefore, we employed the concept of the fuzzy set to define edge-based similarity. In set theory, one of the most solid similarity is following Jaccard’s index [17].

5 $$ {\mathrm{Jaccard}}^{\prime}\mathrm{s}\ \mathrm{index}=\frac{\left|A\cap B\right|}{\left|A\cup B\right|} $$

As a similarity between two fuzzy sets, we employed following definition from previous work similar to Jaccard’s index [18].

6 $$ si{m}_{fuzzy}\left({N}_1,{N}_2\right)=\frac{\left|{N}_1\cap {N}_2\right|}{\left|{N}_1\cup {N}_2\right|}=\frac{\left|\min \left[{\mu}_{N_1}(x),{\mu}_{N_2}(x)\right]\right|}{\left|\max \left[{\mu}_{N_1}(x),{\mu}_{N_2}(x)\right]\right|} $$

where ${\mu}_{N_1}(x),{\mu}_{N_2}(x)$ are degrees of membership for network 1 and network 2. Fig. 2b shows the example of fuzzy set similarity.

### Validation of similarity measures

**Fig. 3:**
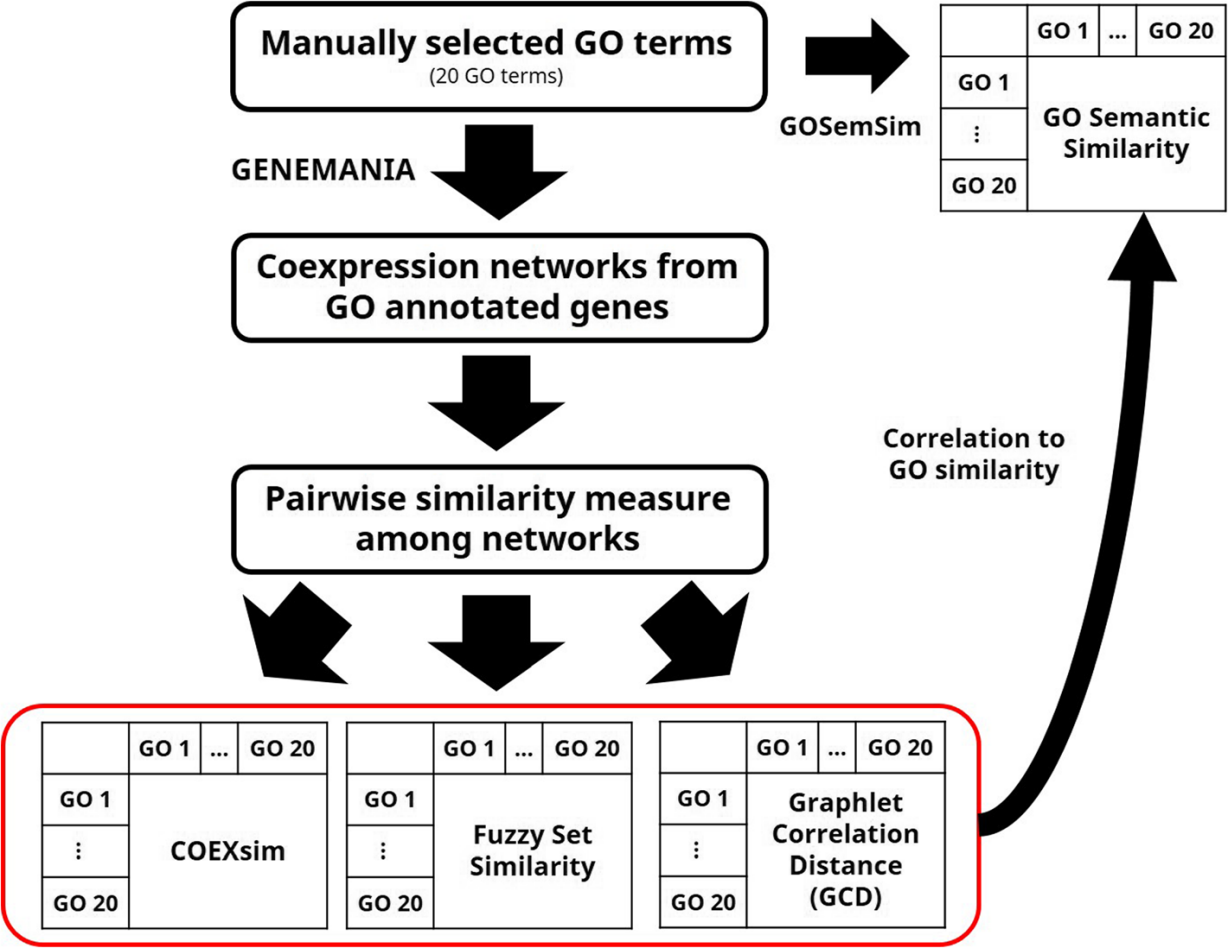
Validation Framework for Coexpression Network Similarity Measures

Since this study is a first attempt to apply network similarity to coexpression analysis, we performed validation of COEXsim and fuzzy set similarity for coexpression networks. For validation, we devised an experimental framework in Fig. 3. As a validation dataset, we selected 20 Gene Ontology (GO) terms [19] and we computed GO semantic similarity among them as a gold standard set because GO semantic similarity reflects information of manually curated gene ontology. As a GO semantic similarity, we employed Schlicker’s method that utilizes information content (IC) to gene ontology and it reflects the relationship of two terms in ontology structure [20]. We used GOSemSim R package to measure GO semantic similarity [21].

Then we constructed coexpression networks of each GO term from GO annotated genes using GENEMANIA [22]. We used GO annotated genes as seed genes and set GENEMANIA to use reported coexpression networks and find a maximum of 150 connected genes from seed genes. As a result, we constructed 20 coexpression networks for GO terms. Detailed GO terms and network statistics are in Additional file 1.

As a previous method to compare, we employed network comparison methods for other biological networks. We did not select alignment-based method because exact matching can replace it as we stated in background section so we selected graphlet-based method, Graphlet Correlation Distance (GCD) due to its novelty (after 2013), and citations (citations > 20) [23]. GCD utilizes information of correlation between each graphlet and define distance as Euclidean distance between graphlet correlation matrix of two networks. We used 73 1~ 3 nodes graphlet for GCD measures and transformed distance to similarity by the following formula.

7 $$ si{m}_{GCD}=\frac{\Big( GCD-\max (GCD)}{\max (GCD)} $$

Then, we measured pairwise similarity among networks by COEXsim, fuzzy set similarity and GCD and computed Spearman correlation coefficient between similarity profile of each method and GO semantic similarity to quantify the validation result.

### Evaluation of similarity threshold

In this study, we had to find ‘similar’ module pairs between HD samples and aging samples. Therefore, we decided to evaluate the threshold that divides ‘similar modules’ and ‘dissimilar modules’. For evaluation, we selected ‘similar group’ and ‘control group’ then we compared two similarities of two groups.

As a similar group, we manually selected two groups that five GO terms related to innate immunity and five GO terms related to angiogenesis. As a control group, we selected five GO terms that are known as not related to innate immunity or angiogenesis. We selected GO terms that have at least 50 annotated genes to provide sufficient seed genes for GENEMANIA. Then, we constructed coexpression networks of each of 15 GO terms from GO annotated genes using GENEMANIA. Detailed GO terms and network statistics are in Table 1.

**Table 1:** Selected GO Terms for Threshold Evaluation and Network Statistics

Gene Ontology ID	Name	# of Nodes	# of Edges
Innate immunity group			
GO:0002228	natural killer cell mediated immunity	147	144,859
GO:0002718	regulation of cytokine production involved in immune response	148	103,074
GO:0034121	regulation of toll-like receptor signaling pathway	150	126,327
GO:0034340	response to type I interferon	148	52,985
GO:0060333	interferon-gamma-mediated signaling pathway	148	154,173
Angiogenesis group			
GO:0002040	sprouting angiogenesis	148	77,037
GO:0007229	integrin-mediated signaling pathway	149	86,185
GO:0045765	regulation of angiogenesis	244	46,229
GO:0048010	vascular endothelial growth factor receptor signaling pathway	149	59,105
GO:0048013	ephrin receptor signaling pathway	148	49,062
Control group			
GO:0007632	visual behavior	149	26,563
GO:0016209	antioxidant activity	147	32,578
GO:0032922	circadian regulation of gene expression	150	32,855
GO:0046365	monosaccharide catabolic process	149	49,308
GO:1900076	regulation of cellular response to insulin stimulus	146	18,655

We measured pairwise similarity among 10 networks (five similar group + five control group) by COEXsim and fuzzy set similarity. Among 100 measured values, we defined that 25 values from within similar group pairs (in short, ‘similar group pairs’) are similarity of the similar group and other values are similarity of the dissimilar group (in short, ‘other pairs’) and we computed median COEXsim and fuzzy set similarity of two groups. We performed these procedures twice for innate immunity group and angiogenesis group. Then, we evaluated threshold for each similarity as an average of two median similarities of similar group pairs.

For disease module analysis, we selected HD – aging module pairs that exceed both COEXsim and fuzzy set similarity thresholds as ‘similar modules’.

### Disease datasets

To identify co-expression modules which were associated with HD and normal brain aging, the publicly available RNA-Seq raw data (FASTQ) files with accession number SRP051844 1 were downloaded from the NCBI short read archive database [24]. The data set consists of RNA-Seq reads from the frontal cortex of 20 cases with Huntington’s disease and 49 normal controls [25].

### Coexpression module extraction

Quality control of the raw sequence data, mapping the RNA-seq reads, and quantifying the mapped reads were performed as previously described [26]. To identify the potential confounding effects in the RNA-Seq data for the HD study, we used surrogate variable analysis (SVA) [27].

For the normal aging study, we first divided the RNA-Seq data into three age groups; young: ≤44, middle: 45–74 and old: ≥ 75, as previously described [28]. The age groups were used as the variable of interest then the surrogate variables were obtained using the SVA package [27]. Then the standardized residuals from the linear regression including the surrogate variables were used to generate gene co-expression networks using WGCNA [29]. To construct a weighted co-expression network we selected the power for which scale-free topology fitting index (R2) is ≥0.9 [30]. Correlation analyses were performed between co-expression modules and traits such as diagnosis, age and descriptive variables to identify modules that were associated with schizophrenia disease status, age and/or confounding factors. To adjust for multiple testing when we performed the correlation analyses, we used the MPTCorr.r package [31] as previously described [26]. We used a trait as a criterion variable and the eigengene values in all modules as multiple predictor variables. Adjusted *p*-values less than 0.05 were considered significant.

## Results

### Validation of similarity measures

To show the validity of COEXsim and fuzzy set similarity, we computed COEXsim and fuzzy set similarity for GO term related coexpression networks then compared them to GO semantic similarity. We measured COEXsim, fuzzy set similarity and GO semantic similarity of 400 network pairs from 20 GO terms. Then, we computed Spearman’s rank correlation coefficient of COEXsim and fuzzy set similarity to GO semantic similarity because scales of three similarities are different (Table 2).

**Table 2:** Correlation of COEXsim and Fuzzy Set Similarity to GO Semantic Similarity

	COEXsim	Fuzzy set similarity	GCD
Spearman Correlation Coefficient	0.55397	0.52450	0.26712
Statistical Significance (*p*-value)	1.5000× 10^−33^	1.1880 ×10^−29^	5.80710×10^−8^

Note that null hypothesis for statistical significance is that the similarity is not correlated to GO semantic similarity

From the result, we examined that both COEXsim and fuzzy set similarity show correlation coefficients over 0.5 with the strong significance of correlation (*p*-value ≈ 10^− 29^). In addition, COEXsim and fuzzy set similarity show higher performance than previous network comparison method, GCD. Therefore, we conclude that both COEXsim and fuzzy set similarity are consistent to GO semantic similarity that reflects expert’s knowledge.

### Evaluation of similarity threshold

We tried to evaluate the minimum similarity of ‘similar module pairs’ to select module pairs. We compared similarity profiles between similar group and control group. We prepared two similar groups: innate immunity group and angiogenesis group so we evaluated thresholds twice separately.

**Fig. 4:**
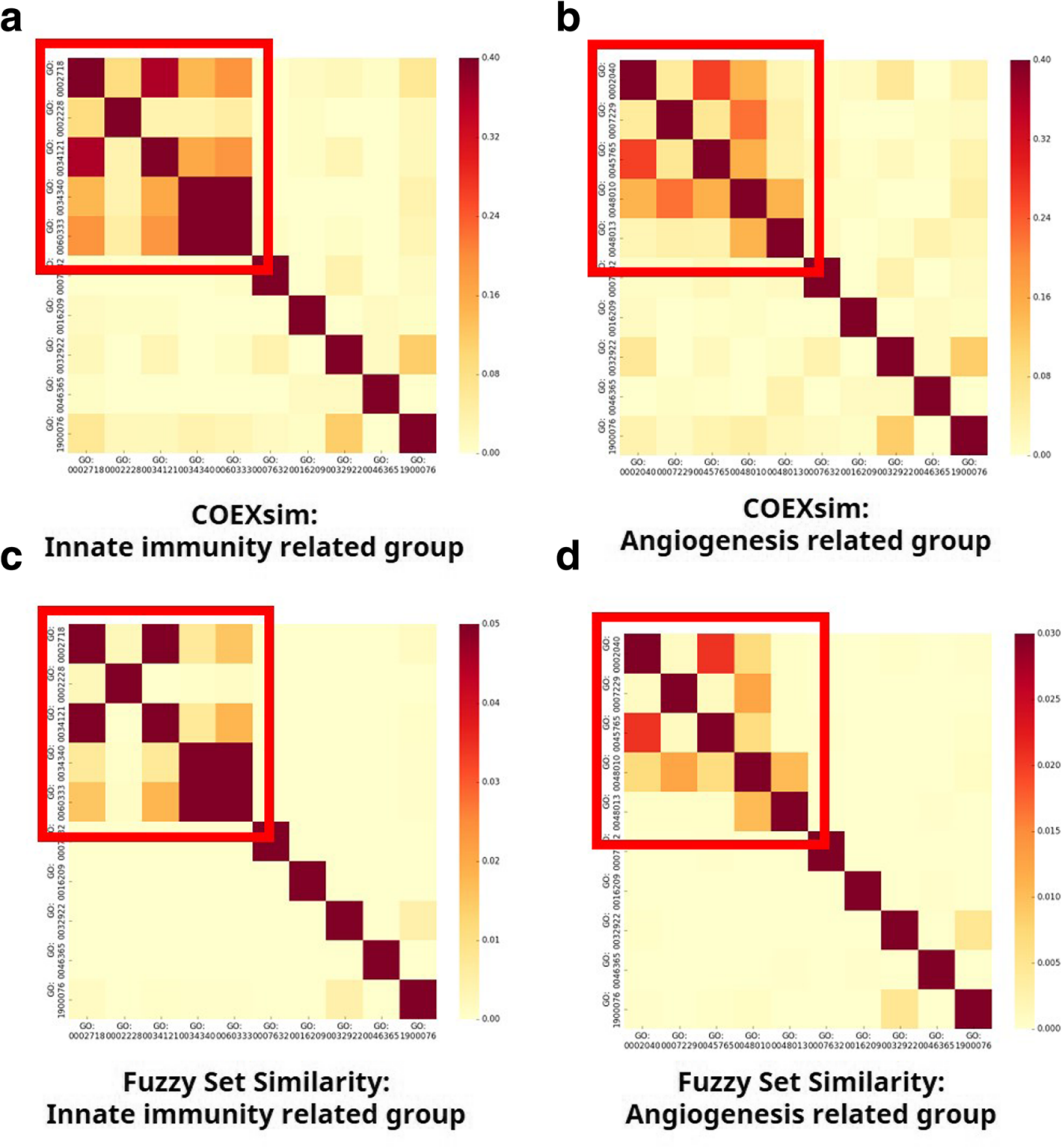
Similarity Measure for Similar Groups and Control Groups. These heatmaps show similarity difference between similar group pairs and other pairs. Redbox indicates similar group pairs. We performed two experiments using different groups for each similarity. COEXsim for (**a**) innate immunity group and (**b**) angiogenesis group. Fuzzy set similarity for (**c**) innate immunity group and (**d**) angiogenesis group

As shown in Fig. 4, both COEXsim and fuzzy set similarity show significantly higher values in similar group pairs (red boxes of each heatmap) than other pairs from both experiments. To evaluate thresholds, we had to determine the representative value of similar group pairs. Therefore, we computed the median of similar group pairs and other pairs because distributions of two similarities are not even.

The result in Table 3 shows clearly that both similarities discriminate similar group pairs and other pairs. Median COEXsim of similar group pairs are around 10-times higher than that of other pairs and median fuzzy set similarity of similar group pairs are around 40-times higher than that of other pairs for both experiments. From this result, we determined average median similarity from both experiments as thresholds of similar module pairs. Therefore, we decided to select coexpression module pairs that have both of COEXsim > 0.1288 and fuzzy set similarity > 0.0055 as ‘similar module pairs’ in disease data analysis.

**Table 3:** Median Similarity Comparison between Similar Group Pairs and Other Pairs

	COEXsim			Fuzzy set similarity		
	Innate immunity	Angiogenesis	Average	Innate immunity	Angiogenesis	Average
Similar group pairs	0.15200	0.10554	0.12877	0.00720	0.00389	0.00554
Other pairs	0.01356	0.01355	0.01355	0.00012	0.00015	0.00013

Note that similar group pairs are similarity between two networks in similar group. COEXsim and Fuzzy set similarity are separately measured and two similar groups (innate immunity and angiogenesis) are used separately

### Analysis of Huntington’s disease and brain aging data

We generated 15 co-expression networks using the RNA-Seq data from frontal cortex of the HD cases and normal controls. Of the 18 co-expression modules, eight modules were significantly associated with HD (all adjusted *p*-values < 0.05, Additional file 5A). Six of the modules positively correlated with HD, indicating that expression levels were upregulated in the frontal cortex of the HD cases as compared to controls. On the other hand, two modules were negatively associated with HD. We also generated 20 co-expression networks using the RNA-Seq data from frontal cortex of the normal controls only. While five modules were significantly correlated with age, three modules were negatively correlated with age (all adjusted *p*-values < 0.05, Additional file 5B).

**Fig. 5:**
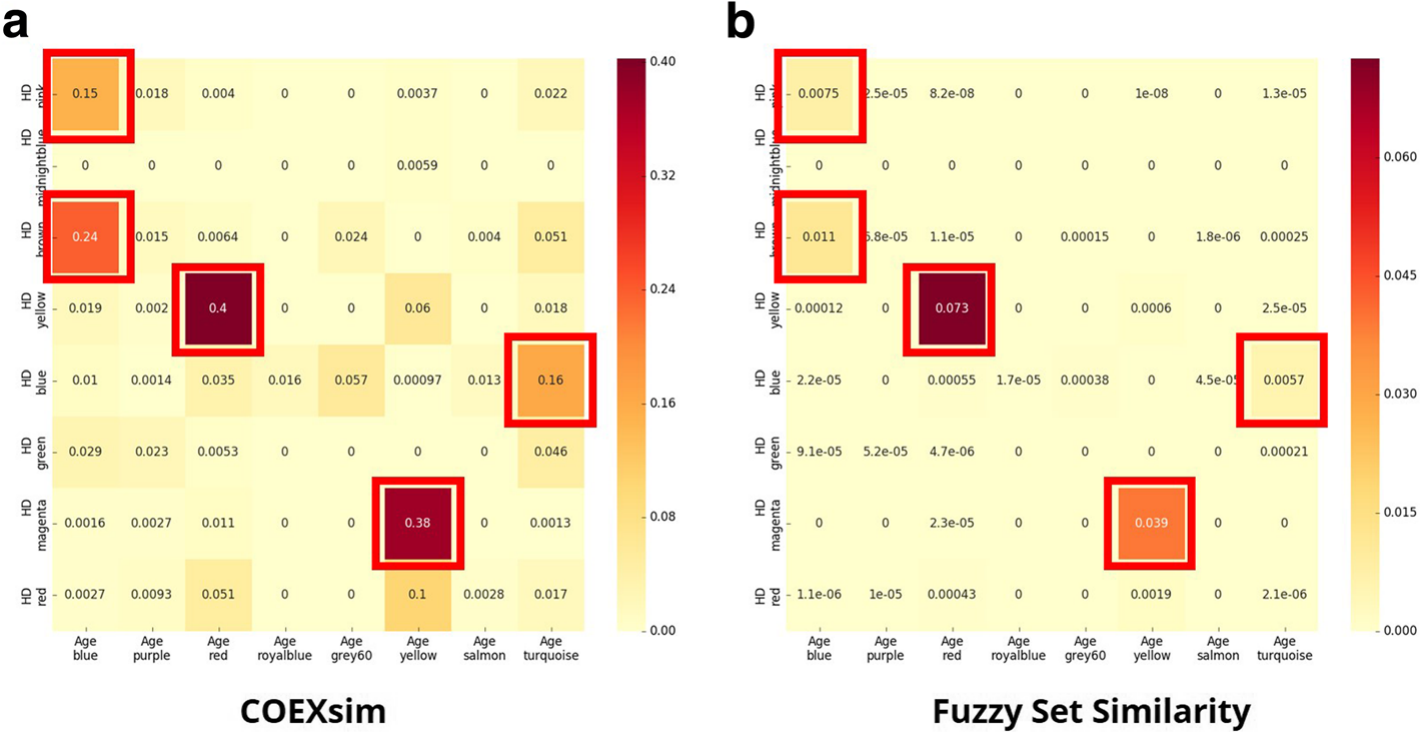
Module Similarity between HD-specific Coexpression Modules and Aging-related Coexpression Modules. These heatmaps show (**a**) COEXsim profile and (**b**) fuzzy set similarity profile between HD-specific modules and aging-related modules. Red boxes indicates selected HD-aging module pairs

We then compared the modules that were associated with HD to the modules that were significantly correlated with normal aging using the COEXsim and fuzzy set similarity to identify coexpression networks that may be common to both HD and normal brain aging (Fig. 5). Using similarity thresholds determined from preceding section, we identified five similar HD-aging coexpression module pairs (Table 4).

**Table 4:** Five Selected HD-Aging Coexpression Module Pairs

HD-specific module	Aging-related module	COEXsim	Fuzzy set similarity
HD-yellow	Age-red	0.40267	0.07254
HD-magenta	Age-yellow	0.37721	0.03934
HD-brown	Age-blue	0.23878	0.01142
HD-blue	Age-turquoise	0.16258	0.00569
HD-pink	Age-blue	0.15155	0.00749

From five similar module pairs, the two most similar pairs are enriched with known neurodegenerative disease mechanisms. HD-yellow module and Age-red module pair showed the highest similarity score (COEXsim: 0.40267, Fuzzy set similarity: 0.07254). These modules were positively associated with HD and normal brain aging, respectively. Genes related to cell signalling, brain development and cell death significantly enriched in the common genes (Additional file 6A). HD-magenta module and Age-yellow module pair showed high similarity score (COEXsim: 0.37721, Fuzzy set similarity: 0.03934). These modules were also positively associated with HD and normal brain aging, respectively. Genes related to immune and inflammation response significantly enriched in the common genes (Additional file 6B).

## Discussion

Aging is known to be a risk factor for several neurodegenerative diseases [32, 33]. However, common molecular networks between HD and normal aging is not known. We therefore explored common coexpression networks between HD and normal brain aging using the two similarity measures that we proposed in this study. In our comparison analysis, HD_yellow module and Age_red module pair and HD_magenta module and Age_yellow module pair showed high similarity scores and the four modules were positively associated with HD and normal brain aging, respectively. The results suggest that up-regulated cell signalling related cell death and immune/ inflammation response may be the common molecular mechanisms in the pathophysiology of HD and normal brain aging in the frontal cortex.As a methodological issue, how to compare coexpression networks from different species is important issue. To apply the method in this study to different species, two networks should be mapped to same species network. We suggest matching two networks by using orthologous genes that maximize the size of common subnetwork by iteration.

## Conclusions

In this study, we proposed similarity measures for quantitative coexpression analysis, COEXsim and fuzzy set similarity. Two similarities utilize gene and their interaction information, respectively. To show validity of two measures, we compared similarity profiles of each method to GO semantic similarity. From the result, we showed that our two measures have superior performance for coexpression network than previous graphlet-based method. Then, we compared similarity profiles between similar network groups and other network groups and evaluated thresholds of two similarities to determine similar coexpression pairs. We applied two similarities to HD and brain aging data and we quantitatively compared HD-specific coexpression modules and aging-related coexpression modules. As a result, we identified five HD-aging module pairs and two of these modules are enriched to the known pathology of neurodegenerative diseases such as brain development, cell death, and immune response.

## Additional files

Additional file 1: Selected GO Terms for Validation and Network Statistics. GO ID, name, number of nodes and number of edges of selected GO terms are included in the file. (XLS 33 kb)

Additional file 2: Similarity Profiles among 20 Coexpression Networks for Validation. The file contains similarity profiles among 20 coexpression networks used for validation. Similarity profiles from GO semantic similarity, COEXsim, fuzzy set similarity and GCD are included in each sheet of the file. (XLS 56 kb)

Additional file 3: Similarity Profiles for Threshold Evaluation. The file contains similarity profiles of two network groups used for threshold evaluation. First and second sheets are similarity profiles of innate immunity group from COEXsim and fuzzy set similarity. Third and last sheets are similarity profiles of angiogenesis group from COEXsim and fuzzy set similarity. (XLS 34 kb)

Additional file 4: Similarity Profiles between HD-specific Modules and Aging-related Modules. The file contains similarity profiles between HD-specific modules and aging-related modules. Two sheets are similarity profiles from COEXsim and fuzzy set similarity, respectively. (XLS 28 kb)

Additional file 5: Correlation Coefficient between Modular Expression and Phenotypes. (A) First sheet contains correlation coefficient between eigenvalue of each module and HD. (B) Second sheet contains correlation coefficient between eigenvalue and age. (XLS 34 kb)

Additional file 6: GO Term Enrichment Analysis Results for Similar HD-aging module pairs. The file contains enriched GO terms of common genes from similar module pairs. (A) First sheet is the result of HD-yellow and Age-red pair. (B) Second sheet is the result of HD-magenta and Age-yellow pair. (XLS 69 kb)

## Abbreviations

COEXsim: Coexpression-based network similarity
GCD: Graphlet correlation distance
GO: Gene ontology
HD: Huntington’s disease
HTT: Huntingtin
WGCNA: Weighted gene coexpression network analysis
